# Association between Plasma Sestrin2 Levels and the Presence and Severity of Coronary Artery Disease

**DOI:** 10.1155/2020/7439574

**Published:** 2020-06-30

**Authors:** Yoshimi Kishimoto, Masayuki Aoyama, Emi Saita, Yukinori Ikegami, Reiko Ohmori, Kazuo Kondo, Yukihiko Momiyama

**Affiliations:** ^1^Endowed Research Department “Food for Health”, Ochanomizu University, Tokyo, Japan; ^2^Department of Cardiology, National Hospital Organization Tokyo Medical Center, Japan; ^3^Faculty of Regional Design, Utsunomiya University, Tochigi, Japan; ^4^Institute of Life Innovation Studies, Toyo University, Gunma, Japan

## Abstract

**Aims:**

Atherosclerotic disease, such as coronary artery disease (CAD), is recognized to be associated with inflammation and oxidative stress. We investigated the association between CAD and plasma levels of sestrin2 which is one of the stress-inducible antioxidant proteins.

**Methods:**

We measured plasma sestrin2 levels in 304 patients undergoing elective coronary angiography. The severity of CAD was represented as the numbers of >50% stenotic coronary vessels and segments and the severity score.

**Results:**

CAD was found in 175 patients, of whom 73 had 1-vessel (1-VD), 59 had 2-vessel (2-VD), and 43 had 3-vessel disease (3-VD). Plasma sestrin2 levels were significantly higher in 175 patients with CAD than in 129 without CAD (median 16.4 vs. 14.2 ng/mL, *P* < 0.05). A stepwise increase in sestrin2 levels was found depending on the number of >50% stenotic coronary vessels: 14.2 in CAD(-), 15.4 in 1-VD, 17.3 in 2-VD, and 17.7 ng/mL in3-VD (*P* < 0.05). High sestrin2 level (>16.0 ng/mL) was present in 38% of patients with CAD(-), 47% of 1-VD, 66% of 2-VD, and 53% of 3-VD (*P* < 0.005). Sestrin2 levels significantly, but weakly, correlated with the number of >50% stenotic segments and the severity score (rs = 0.12 and rs = 0.13, *P* < 0.05). In the multivariate analysis, sestrin2 levels were a significant factor associated with CAD independent of atherosclerotic risk factors. The odds ratio for CAD was 1.79 (95%CI = 1.09‐2.95) for high sestrin2 level of >16.0 ng/mL (*P* < 0.025).

**Conclusions:**

Plasma sestrin2 levels in patients with CAD were found to be high and to be associated with the severity of CAD. High sestrin2 levels in patients with CAD may reflect a protective response against the progression of CAD.

## 1. Introduction

Atherosclerotic disease, such as coronary artery disease (CAD), is recognized to be a chronic inflammatory disease, and oxidative stress by excessive reactive oxygen species (ROS) contributes to the pathogenesis of atherosclerosis [1, 2]. Endogenous antioxidants may play a protective role against the progression of atherosclerosis [1, 2]. Sestrin2 is a stress-inducible antioxidant protein that was originally identified as a hypoxia-induced gene 95 (Hi95) [3]. Sestrin2 is expressed and secreted mainly by macrophages, T lymphocytes, and endothelial cells [4–6]. Sestrin2 expression was induced by angiotensin II in human umbilical vein endothelial cells (HUVECs), and the knockdown of sestrin2 promoted angiotensin II-induced apoptosis and oxidative stress [7]. Sestrin2 expression was also upregulated in macrophages by oxidized LDL, and the upregulation of sestrin2 reduced cell apoptosis and oxidative stress [4]. Moreover, sestrin2 reduced lipopolysaccharide-induced proinflammatory cytokine release, cell death, and ROS production in macrophages [8]. The knockdown of sestrin2 in HUVECs promoted lipopolysaccharide-induced inflammatory responses, cell death, and ROS production [9]. These evidences thus suggest that sestrin2 has antioxidant and anti-inflammatory properties.

Atherosclerosis is recognized to be associated with inflammation with oxidative stress [1, 2], and sestrin2 is suggested to play a protective role against atherosclerotic disease [10, 11]. Regarding the association between sestrin2 and atherosclerotic disease, one small study [12] recently reported that plasma sestrin2 levels were higher in 44 patients with stable CAD than in 35 patients without CAD. However, in their study, multivariate analysis was not performed in spite of significant differences in atherosclerotic risk factors between patients with and without CAD. In the present study, we investigated the associations between plasma sestrin2 levels and the presence and severity of CAD in 304 patients undergoing elective coronary angiography.

## 2. Methods

### 2.1. Study Patients

We measured plasma sestrin2 levels in 304 consecutive patients who underwent elective coronary angiography for suspected CAD at Tokyo Medical Center from June 2009 to September 2016. Patients with acute coronary syndrome, defined as acute myocardial infarction (AMI) and class III unstable angina at rest by Braunwald's classification [13], were excluded from this study. Patients with a history of percutaneous coronary intervention or cardiac surgery or those with known aortic diseases were also excluded. Since plasma sestrin2 levels in patients with heart failure were reported to be high and to increase from New York Heart Association functional class II to IV [6], patients with a history of heart failure were also excluded from this study. Hypertension was defined as blood pressures of ≥140/90 mmHg or on drugs, and 180 (59%) patients were taking antihypertensive drugs. Hyperlipidemia was defined as an LDL-cholesterol level of >140 mg/dL or on drugs, and 116 (38%) patients were taking statins. Diabetes mellitus (DM) (a fasting plasma glucose (FPG) level of ≥126 mg/dL or on treatment) was present in 83 (27%) patients, and 106 (35%) were smokers (≥10 pack-years). Our study was approved by the institutional ethics committee of our hospital (R07-054/R15-056). After written informed consent was obtained, overnight-fasting blood samples were taken on the morning of the day when coronary angiography was performed.

### 2.2. Measurements of Plasma Sestrin2 and C-reactive Protein (CRP) Levels

Blood samples were collected in EDTA-containing tubes, and the plasma was stored at −80°C. Plasma sestrin2 levels were measured by an enzyme-linked immunosorbent assay (ELISA) with a commercially available kit (ELISA Kit for Sestrin2, Cloud-Clone, TX, USA) at Ochanomizu University according to the manufacturer's instructions. The intra- and interassay coefficients of variation were <10% and <12%, respectively. Plasma high-sensitivity C-reactive protein (hsCRP) levels were also measured by a BNII nephelometer (Dade Behring, Tokyo, Japan).

### 2.3. Coronary Angiography

Coronary angiograms were recorded on a cineangiogram system (Philips Electronics Japan, Tokyo, Japan). CAD was defined as at least one coronary artery having >50% luminal diameter stenosis on angiograms. The severity of CAD was represented as the numbers of >50% stenotic vessels and stenotic segments and the severity score of stenosis. The degree of coronary stenosis in each segment was scored from 0 to 4 points (0, ≤25%; 1, 26%-50%; 2, 51%-75%; 3, 76%-90%; and 4, >90% stenosis), and then, the severity score was defined as the sum of scores of all segments. Coronary artery segments were defined as 29 segments according to the Coronary Artery Surgery Study (CASS) classification. All angiograms were evaluated by a single cardiologist (Y.M.), blinded to the clinical and laboratory data.

### 2.4. Statistical Analysis

Differences between 2 groups were evaluated by unpaired *t*-test for parametric variables, by Mann-Whitney *U* test for nonparametric variables, and by chi-squared test for categorical variables. Differences among ≥3 groups were evaluated by an analysis of variance with Scheffe's test for parametric variables, by Kruskal-Wallis test with Steel-Dwass test for nonparametric variables, and by chi-squared test for categorical variables. Correlations between plasma sestrin2 or hsCRP levels and the severity of CAD were evaluated by Spearman's rank correlation test. To determine the cut-off point of plasma sestrin2 levels for CAD, a receiver-operating characteristic curve was created, and then, the optimal cut-off point was determined to be 16.0 ng/mL as the point where the Youden index was maximum. Regarding the cut-off point of hsCRP levels, the previously reported cut-off point of 1.0 mg/L for CAD was used [14, 15]. A forward stepwise multiple logistic regression analysis was performed to determine the independent association between sestrin2 levels and CAD. All statistical analyses were carried out using the SPSS software package (IBM SPSS version 25, Tokyo, Japan). A *P* value of <0.05 was considered to be statistically significant. The results are presented as the mean ± SD or the median value.

## 3. Results

**Figure 1:**
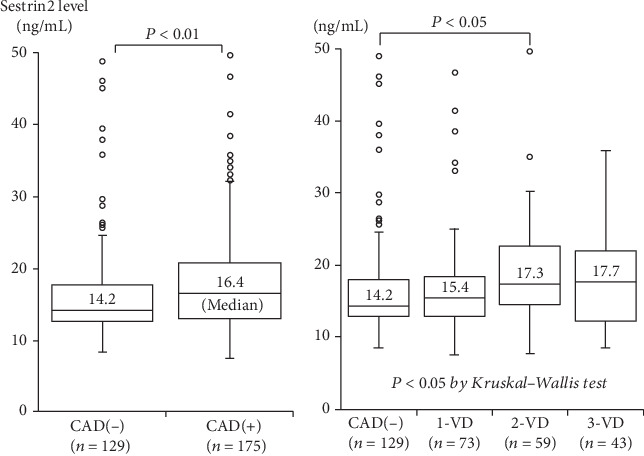
Plasma sestrin2 levels and the presence of CAD or the number of stenotic coronary vessels. Plasma sestrin2 levels were significantly higher in CAD than in CAD(-) (a). Moreover, sestrin2 levels in 4 groups of CAD(-), 1-VD, 2-VD, and 3-VD were 14.2, 15.4, 17.3, and 17.7 ng/mL, respectively, and were the highest in 3-VD (*P* < 0.05 by Kruskal-Wallis test) (b). The central line represents the median, and the box represents the 25th to 75th percentiles. The whiskers represent the lowest and highest values in the 25th percentile minus 1.5 IQR and 75th percentile plus 1.5 IQR, respectively.

Among the 304 study patients, CAD was present in 175 patients (58%) (1-vessel disease (1-VD), *n* = 73; 2-vessel disease (2-VD), *n* = 59; and 3-vessel disease (3-VD), *n* = 43). Compared with 129 patients without CAD, 175 patients with CAD were older and had a male predominance; higher prevalence of hypertension, hyperlipidemia, and DM; and lower HDL-cholesterol levels (Table 1). Plasma hsCRP levels were higher in patients with CAD than in those without CAD (median 0.57 vs. 0.43 mg/L, *P* < 0.01). A stepwise increase in hsCRP levels was found depending on the number of >50% stenotic coronary vessels: 0.43 in CAD(-), 0.54 in 1-VD, 0.57 in 2-VD, and 0.60 mg/L in 3-VD (*P* < 0.02) (Table 1). Notably, plasma sestrin2 levels were significantly higher in patients with CAD than in those without CAD (median 16.4 vs. 14.2 ng/mL, *P* < 0.05) (Figure 1). A stepwise increase in sestrin2 levels was found depending on the number of >50% stenotic vessels: 14.2 in CAD(-), 15.4 in 1-VD, 17.3 in 2-VD, and 17.7 ng/mL in 3-VD and was the highest in 3-VD (*P* < 0.05) (Figure 1). A high sestrin2 level (>16.0 ng/mL) was present in 38% of patients with CAD(-), 47% of 1-VD, 66% of 2-VD, and 53% of 3-VD (*P* < 0.005). Although hsCRP levels correlated with the number of >50% stenotic coronary segments and the severity score (rs = 0.16 and rs = 0.16, *P* < 0.01), sestrin2 levels significantly, but weakly, correlated with the number of >50% stenotic segments and the severity score (rs = 0.12 and rs = 0.13, *P* < 0.05).

Sestrin2 levels also correlated with age (rs = 0.29, *P* < 0.001). To clarify the association between age and sestrin2 levels, the 304 study patients were divided into 2 groups by age: patients aged < 70 yrs (*n* = 145) and ≥70 yrs (*n* = 159). Compared with patients aged < 70 yrs, those aged ≥ 70 yrs had higher sestrin2 levels (17.0 vs. 14.2 ng/mL, *P* < 0.001) and higher prevalence of CAD (65% vs. 50%, *P* < 0.025). Among patients aged < 70 yrs, sestrin2 levels were significantly higher in patients with CAD than in those without CAD (15.4 vs. 13.6 ng/mL, *P* < 0.05), whereas among patients aged ≥ 70 yrs, sestrin2 levels tended to be higher in patients with CAD than in those without CAD (17.2 vs. 15.9 ng/mL) (Table 2).

To elucidate the independent association between plasma sestrin2 levels and CAD, variables (age, gender, hypertension, hyperlipidemia, statin use, DM, smoking, and HDL-cholesterol, hsCRP, and sestrin2 levels) were entered into a multiple logistic regression model. Plasma sestrin2 levels were a significant factor associated with CAD independent of atherosclerotic risk factors. The odds ratio for CAD was 1.79 (95%CI = 1.09‐2.95) for the high sestrin2 level of >16.0 ng/mL (*P* < 0.025) (Table 3).

## 4. Discussion

In the present study, plasma sestrin2 levels were significantly higher in patients with CAD than in those without CAD, and they positively, but weakly, correlated with the severity of CAD, defined as the numbers of >50% stenotic vessels and segments and the severity score. High plasma sestrin2 levels were found to be a significant factor associated with CAD independent of atherosclerotic risk factors.

Regarding the association between sestrin2 and atherosclerotic diseases, Xiao et al. [16] reported that sestrin2 expression was upregulated in aortic specimens from 12 patients with aortic dissection and that plasma sestrin2 levels were higher in 120 patients with aortic dissection than in 40 patients without dissection. In contrast, Chung et al. [17] investigated serum sestrin2 levels and carotid intima-media thickness (IMT) measured by ultrasonography in 194 diabetic patients. However, they found no significant correlation between serum sestrin2 levels and carotid mean IMT.

Regarding the association between sestrin2 levels and coronary atherosclerosis, Ye et al. [12] measured plasma sestrin2 levels in 119 patients with CAD (stable angina (SA), *n* = 44; unstable angina (UA), *n* = 41; and acute myocardial infarction (AMI), *n* = 29) and 35 without CAD. They reported sestrin2 levels to be higher in patients with CAD than in those without CAD and to be much higher in UA and AMI than in SA. However, in their study, any multivariate analysis was not performed in spite of some significant differences in atherosclerotic risk factors between patients with and without CAD. They also reported sestrin2 levels to correlate well with the Gensini score (*r* = 0.46), but this correlation coefficient was analyzed in CAD patients including patients with AMI. Since sestrin2 expression was demonstrated to be upregulated in cardiac macrophages of a mouse MI model [18], our present study excluded patients with AMI. In the present study, we found that plasma sestrin2 levels were significantly higher in 175 patients with CAD than in 129 without CAD and that they were a significant factor associated with CAD independent of atherosclerotic risk factors. We also found that sestrin2 levels correlated with the severity of CAD. However, the correlation between sestrin2 levels and the severity of CAD, defined as the number of stenotic segments and the severity score, was significant but weak (*r* = 0.12 and *r* = 0.13). Moreover, as shown in Figure 1, there was a substantial overlap in sestrin2 levels between patients with and without CAD. Therefore, plasma sestrin2 levels in patients with CAD may reflect not only the degree of coronary atherosclerosis but also that of atherosclerosis in other vascular beds, and sestrin2 levels are unlikely to be a good biomarker for the presence or severity of CAD. Since sestrin2 is considered to have antioxidant and anti-inflammatory properties [4, 7–9], high plasma levels of sestrin2 in patients with CAD may reflect a compensatory response to increased oxidative stress and may be aimed at protecting against the progression of CAD. However, further studies are needed to elucidate the main source and role of high plasma sestrin2 levels in patients with CAD.

Sestrin2 is suggested to be associated with aging and age-related diseases [10, 11], and plasma sestrin2 levels were reported to positively correlate with age [12, 17]. In our study, sestrin2 levels also correlated with age (*r* = 0.29). Compared with patients aged < 70 yrs, those aged ≥ 70 yrs more often had CAD and had higher sestrin2 levels. Moreover, in patients aged < 70 yrs, sestrin2 levels were significantly higher in patients with CAD than in those without CAD. However, in patients aged ≥ 70 yrs, sestrin2 levels tended to be higher in patients with CAD than in those without CAD.

Our study has several limitations. First, in our study, angiography was used to evaluate coronary atherosclerosis. Angiography cannot visualize plaques and only shows lumen characteristics. However, intravascular ultrasound (IVUS) or optical coherence tomography (OCT), which can visualize coronary plaques, was not always performed in our patients. Second, sestrin2 was reported to be expressed and secreted by various cells, mainly by macrophages, T lymphocytes, and endothelial cells [4–6]. However, since we did not measure sestrin2 levels in the coronary sinus, our study did not provide any information about the main sources of sestrin2 in patients with CAD. Third, our study was cross-sectional in nature and was unable to establish causality, since it only depicted some associations and proposed some hypotheses. Finally, as in our previous studies [19, 20], the present study was performed in Japanese patients undergoing coronary angiography, who are generally considered to be a highly selected population at high risk for CAD. Our results therefore may not be applicable to the general or other ethnic populations.

In conclusion, plasma sestrin2 levels in patients with CAD were found to be high and to be associated with the severity of CAD. Our results suggest that high sestrin2 levels in patients with CAD may reflect a protective response against the progression of CAD.

## Figures and Tables

**Table 1:** Clinical characteristics and plasma sestrin2 levels of patients with and without CAD.

	CAD(-) (*n* = 129)	*Pvalue CAD(-) vs. CAD*	CAD (*n* = 175)	1-VD (*n* = 73)	2-VD (*n* = 59)	3-VD (*n* = 43)	*Pvalue among 4 groups*
Age (years)	67 ± 10	*<0.02*	69 ± 10	68 ± 10	69 ± 10	72 ± 8	*<0.05*
Gender (male)	77 (60%)	*<0.01*	131 (75%)	55 (74%)	42 (71%)	35 (81%)	*<0.025*
BMI (kg/m^2^)	23.9 ± 4.6	*NS*	23.7 ± 3.4	24.0 ± 3.5	23.9 ± 3.1	23.1 ± 3.7	*NS*
Hypertension	76 (59%)	*<0.001*	136 (78%)	55 (75%)	44 (75%)	37 (86%)	*<0.005*
SBP (mmHg)	128 ± 21	*<0.02*	134 ± 18	133 ± 17	137 ± 21	132 ± 16	*<0.05*
Diabetes mellitus	21 (16%)	*<0.001*	62 (35%)	19 (26%)	24 (41%)	19 (44%)	*<0.001*
HbA1c (%)	5.9 ± 0.6	*<0.001*	6.3 ± 0.9	6.1 ± 0.7	6.5 ± 1.1	6.4 ± 1.0	*<0.001*
Smoking	39 (30%)	*NS*	67 (38%)	29 (40%)	24 (41%)	14 (33%)	*NS*
Hyperlipidemia	53 (41%)	*<0.01*	100 (57%)	41 (56%)	34 (58%)	25 (58%)	*NS*
Statin	37 (29%)	*<0.01*	79 (45%)	32 (44%)	26 (44%)	21 (49%)	*<0.05*
LDL-C (mg/dL)	112 ± 28	*NS*	114 ± 33	111 ± 35	115 ± 31	118 ± 29	*NS*
HDL-C (mg/dL)	59 ± 15	*<0.001*	52 ± 13	55 ± 14	50 ± 12	48 ± 13	*<0.001*
hsCRP (mg/L)	0.43 [0.22, 0.90]	*<0.01*	0.57 [0.32, 1.41]	0.54 [0.30, 1.04]	0.57 [0.36, 1.32]	0.60 [0.39, 1.85]	*<0.02*
hsCRP > 1.0 mg/L	30 (23%)	*NS*	55 (31%)	19 (26%)	19 (32%)	17 (40%)	*NS*
Sestrin2 levels (ng/mL)	14.2 [12.8, 17.9]	*<0.05*	16.4 [13.0, 20.7]	15.4 [12.9, 18.3]	17.3 [14.5, 22.5]	17.7 [12.1, 22.0]	*<0.05*
Sestrin2 > 16.0 ng/mL	49 (38%)	*<0.01*	96 (55%)	34 (47%)	39 (66%)	23 (53%)	*<0.005*

Data represent the mean ± SD or the number (%) of patients, with the exception of hsCRP and sestrin2 levels which are presented as the median value and interquartile range. BMI: body mass index; SBP: systolic blood pressure; LDL-C: low-density lipoprotein cholesterol; HDL-C: high-density lipoprotein cholesterol.

**Table 2:** Clinical characteristics and plasma sestrin2 levels in the subgroups of patients aged < 70 years and ≥70 years.

	Age < 70			Age ≥ 70		
	CAD(-) (*n* = 73)	*CAD(-) vs. CAD*	CAD (*n* = 72)	CAD(-) (*n* = 56)	*CAD(-) vs. CAD*	CAD (*n* = 101)
Age (years)	60 ± 8	*NS*	60 ± 7	76 ± 4	*NS*	76 ± 4
Gender (male)	45 (62%)	*<0.025*	58 (81%)	32 (57%)	*NS*	73 (71%)
Hypertension	39 (53%)	*<0.01*	55 (76%)	37 (66%)	*NS*	81 (79%)
Diabetes mellitus	13 (18%)	*<0.025*	27 (38%)	8 (14%)	*<0.025*	35 (34%)
Smoking	26 (36%)	*NS*	33 (46%)	13 (23%)	*NS*	34 (33%)
Hyperlipidemia	29 (40%)	*<0.025*	43 (60%)	24 (43%)	*NS*	57 (55%)
Statin	17 (23%)	*<0.01*	33 (46%)	20 (36%)	*<0.01*	46 (45%)
LDL-C (mg/dL)	113 ± 29	*NS*	116 ± 35	110 ± 27	*NS*	113 ± 31
HDL-C (mg/dL)	59 ± 16	*<0.001*	50 ± 12	58 ± 13	*<0.02*	53 ± 14
hsCRP (mg/L)	0.40 [0.21, 0.94]	*<0.05*	0.58 [0.30, 1.52]	0.46 [0.26, 0.87]	*NS*	0.57 [0.32, 1.31]
hsCRP > 1.0 mg/L	18 (25%)	*NS*	24 (33%)	12 (21%)	*NS*	31 (30%)
Sestrin2 levels (ng/mL)	13.6 [12.5, 16.8]	*<0.05*	15.4 [12.8, 20.0]	15.9 [13.6, 22.3]	*NS*	17.2 [13.3, 21.4]
Sestrin2 > 16.0 ng/mL	21 (29%)	*<0.05*	34 (47%)	28 (50%)	*NS*	62 (60%)

Data represent the mean ± SD or the number (%) of patients, with the exception of hsCRP and sestrin2 levels which are presented as the median value and interquartile range.

**Table 3:** Factors associated with CAD (multiple logistic regression analysis of the 304 study patients).

	Odds ratio	(95% CI)	*Pvalue*
Male gender	2.19	(1.25-3.83)	*<0.01*
Hypertension	1.88	(1.09-3.24)	*<0.025*
Hyperlipidemia	1.99	(1.17-3.40)	*<0.02*
Diabetes mellitus	2.15	(1.18-3.90)	*<0.02*
High sestrin2 level (>16.0 ng/mL)	1.79	(1.09-2.95)	*<0.025*

The dependent variable was the presence of CAD. The analysis included age, gender, hypertension, hyperlipidemia, statin use, diabetes mellitus, smoking, and HDL-cholesterol (<40 mg/dL), hsCRP (>1.0 mg/L), and sestrin2 (>16.0 ng/mL) levels.

## Data Availability

The data that support the findings of this study are available from the corresponding author on reasonable request.
